# Orthopaedic Simulation of a Morton’s Extension to Test the Effect on Plantar Pressures of Each Metatarsal Head in Patients without Deformity: A Pre-Post-Test Study

**DOI:** 10.3390/diagnostics13193087

**Published:** 2023-09-28

**Authors:** Anna Sánchez-Serena, Marta Elena Losa-Iglesias, Ricardo Becerro-de-Bengoa-Vallejo, Ángel Morales-Ponce, Alfredo Soriano-Medrano, Eduardo Pérez-Boal, Jessica Grande-del-Arco, Israel Casado-Hernández, Eva María Martínez-Jiménez

**Affiliations:** 1 Nursing Department, Faculty of Nursing, Physiotherapy and Podiatry, Universidad Complutense de Madrid, 28040 Madrid, Spain; annasa02@ucm.es (A.S.-S.); ribebeva@enf.ucm.es (R.B.-d.-B.-V.); alfresor@ucm.es (A.S.-M.); jessgran@ucm.es (J.G.-d.-A.); evamam03@ucm.es (E.M.M.-J.); 2 Department of Nursing and Stomatology, Faculty of Health Sciences, King Juan Carlos University, Alcorcon Campus, 28922 Madrid, Spain; marta.losa@urjc.es (M.E.L.-I.); angel.morales@urjc.es (Á.M.-P.); 3 Department of Nursing and Physiotherapy, Faculty of Health Sciences, Universidad de León, 24004 León, Spain

**Keywords:** pressure, foot, pressure platform, metatarsalgia, Morton’s extension, orthopaedics

## Abstract

Background: the area beneath the metatarsal heads is a common location of foot pain, which is often associated with high plantar pressures. The aim of this study was to determine the effect of the application of a Morton’s extension on the pressure in the metatarsal bones of the foot using a pressure platform. Methods: twenty-five subjects without musculoskeletal pathology were selected for this study, and an experiment was conducted with them as the subjects, before and after application of a Morton’s extension. The foot regions were divided into the forefoot (transversely subdivided into six areas corresponding to the first, second, third, fourth, and fifth metatarsal heads, and the hallux), midfoot, and rearfoot, and then the maximum and average pressures exerted at each region were measured before and after placing a Morton’s extension. Main findings: we found a pressure reduction, with a *p*-value less than (*p* < 0.05), in the head of the second and third metatarsals in statics and dynamics. Conclusions: we can conclude that the Morton’s extension produces a variation in plantar pressures on the lesser metatarsals. The application of a Morton’s extension may be beneficial for the management of forefoot pathology. This study will help clinicians consider various tools to treat forefoot disorders. NCT05879094 (ClinicalTrial.gov (accessed on 18 May 2023)).

## 1. Introduction

Central metatarsalgia, one of the most prevalent issues in orthopaedic clinical practice, refers to a painful condition localised in the plantar forefoot region between the second and fourth metatarsal heads and/or the metatarsophalangeal joint [1]. Approximately 10% of the general population has experienced some form of pain in the metatarsal region during their lifetime, with most cases occurring in females [2]. This condition should not be considered a distinct diagnosis; it is a symptom with several potential contributing factors [2]. It can arise from various underlying disorders but is most commonly linked to mechanical alterations of the forefoot, such as claw toe, insufficiency of the great toe, or hallux valgus [3].

Additional primary causes of metatarsalgia include a disproportionate length of the second or third metatarsal, congenital deformities of the metatarsal heads, tightness in the gastrocnemius muscles or triceps, fixed equinus of the foot, pes cavus, and any hindfoot abnormality that leads to excessive loading of the forefoot [4].

Abnormal foot function can lead to elevated plantar foot pressures and subsequent foot pain and disability [5]. Musculoskeletal disorders and deformities are closely associated with increased forefoot pressure [6]. Information regarding plantar pressure forces is commonly utilised to identify and analyse various foot disorders and impairments [7]. Elevated plantar pressure can be mitigated through offloading or load-redistributing strategies involving therapeutic insoles and/or footwear options [8]. Although the specific mechanisms by which foot orthoses provide benefits remain unclear, studies have demonstrated their capacity to alter plantar pressure distribution, enhance sensory feedback, influence muscle activity, and impact the kinematics of the lower limb during walking and running [9]. Conclusive evidence supports the effectiveness of foot orthoses in alleviating symptoms in specific types of foot-related pathologies, such as plantar fasciitis, rheumatoid arthritis, and foot pain (e.g., metatarsalgia). Additionally, they contribute to improved comfort [10].

Owings et al. [11] optimised pressure relief using plantar pressure and foot shape data to guide the metatarsal bar shape and placement design. Studies by Hayda et al. and Holmes et al. demonstrated that the placement of metatarsal pads can effectively reduce pressure beneath the metatarsal heads in healthy individuals [12,13].

In a systematic review, Arias-Martin et al. concluded that the utilisation of custom-made foot orthoses led to an improvement in the level of forefoot pain in conditions such as rheumatoid arthritis, hallux abductus valgus, and secondary metatarsalgia, attributed to an increase in sole pressures [14].

However, limited studies have investigated the effects of orthopaedic devices on plantar pressures using the Morton’s extension device. Morton’s extension, an element positioned beneath the anterior part of the foot orthosis under the first ray, extends from the first metatarsal head to the proximal phalanx of the hallux [15]. Constructed from a firm or rigid orthopaedic material, the Morton’s extension extends below the great toe, aiding in the reduction of the dorsiflexed position of the first ray in the sagittal plane [15]. Proper dorsiflexion movement of the metatarsophalangeal joint during motion is essential for stabilising the foot in the final contact phase of gait and ensuring effectiveness [16]. Failure to achieve gait stabilisation and inefficiency across different gait phases gives rise to secondary pathologies, and increases energy expenditure and functional costs during gait, disrupting the muscle–ligament integrity of bodily structures [17]. Morton’s extensions have found use in orthopaedic practice to address conditions such as hallux limitus [18] or hallux rigidus [19], both of which are associated with incorrect positioning of the first ray’s axis in the sagittal plane.

Hence, this study aimed to assess the effects of a Morton’s extension applied to the first metatarsophalangeal joint area in individuals without foot abnormalities. An analysis and measurement system was employed to gauge the pressure exerted on the forefoot, midfoot, and hindfoot to detect any changes. Given the distinctiveness of our research and the available literature, making direct comparisons with other studies proves challenging [20].

Our hypothesis posited that using a Morton’s extension would influence pressure distribution in the forefoot by reducing pressure on the lesser metatarsals. The principal objective of this investigation was to establish that the Morton’s extension effectively alleviates pressure from the minor metatarsals.

## 2. Materials and Methods

### 2.1. Design and Sample

A descriptive and observational study was carried out.

### 2.2. Participants

Using G*Power^®^ 3.1.9.7 software, we computed the necessary sample size to achieve the study’s objective. The aim was to observe differences before and after applying Morton’s stretch. Examining a study with a similar methodology conducted by researchers investigating the acute effects of intermittent stretching on plantar pressures during standing, it is noted that these authors discovered a reduction in the maximum rearfoot pressure (measured in kPa) following stretching, decreasing from 106.24 ± 20.89 to 87.45 ± 22.28 (*p* = 0.004) [21,22]. Therefore, employing the sample size calculation for the related samples difference test (same group before and after an intervention), with a two-tailed hypothesis at a statistical confidence level of 95%, an alpha error probability of 0.05, and a power of 0.80, a sample size of 18 subjects was determined.

Considering the potential for attrition, 25 subjects were enlisted for the study. The sample was assembled using a consecutive sampling method, employing a successive and non-randomised simple approach.

Participation selection and inclusion criteria were as follows: (1) no history of trauma to the foot; (2) the presence of at least 10° of dorsiflexion at the ankle with the knee fully dorsiflexed; (3) unrestricted motion of the functional subtalar joint of 30°; (4) unrestricted motion along the longitudinal axis of the midtarsal joint of 15°; (5) unrestricted non-weight-bearing motion of the first ray of at least 8 mm; (6) greater than 50° of dorsiflexion of the hallux to the first metatarsal bisection during non-weight-bearing; (7) age greater than 18 years and younger than 60 years; (8) at the time of data collection, no lower limb dysfunction or chronic injury; and (9) no evidence of a non-fixed deformity at the first MTP and first metatarsal cuneiform joints.

The exclusion criteria of subjects were: (1) plantar corns and calluses, (2) hallux valgus and lesser toe deformities, (3) diabetes, and (4) lower limb dysfunction [15].

### 2.3. Ethical Considerations

This research received approval from the ethics committee of CEIC Hospital Clínico San Carlos (code: 20/235-E). Prior to the commencement of the study, all participants provided their informed consent by signing the relevant form. The principles outlined in the Helsinki Declaration and all human experimentation regulations were duly adhered to [23].

### 2.4. Procedure

A foot pressure measurement device (FOOT TECHNOLOGY, S.L. Barcelona, España, www.medicimage.info (accessed on 12 February 2020) was employed to assess pressure distribution across the sole. The device possessed the following specifications: an active area of 400 × 400 mm, dimensions of 475 × 450 × 25 mm, thickness of 4 mm, pressure range from 10 kPa to 1200 kPa, and a frequency of 300 MHz. This instrument was equipped with 6024 sensors.

Static foot pressure was assessed by positioning the tips of both feet in alignment with the vertical and horizontal lines of the foot pressure measuring plate. Participants were instructed to face forward, avoid wearing shoes, and assume a double-leg stance on the sensor plate for 25 s [24]. This process was repeated for three measurement trials [25].

The walking measurement procedure adhered to the approach advocated by Palisanol et al. [26]. Participants commenced from a designated starting position, marked where their fifth step engaged the sensor plate. Walking was performed barefoot at a natural, comfortable pace. Each trial required the entire foot length to be captured on the sensor plate to ensure accurate data collection. After two preliminary trials for each lower limb to familiarise participants, three valid trials were subsequently recorded for each leg alternatingly [25].

Adhering to a consistent protocol administered by the same specialist across all study participants, a 5-mm EVA Morton’s extension was uniformly affixed from the first metatarsal head to the proximal phalanx of the hallux [15]. A paper bandage secured this component in place [27].

**Figure 1:**
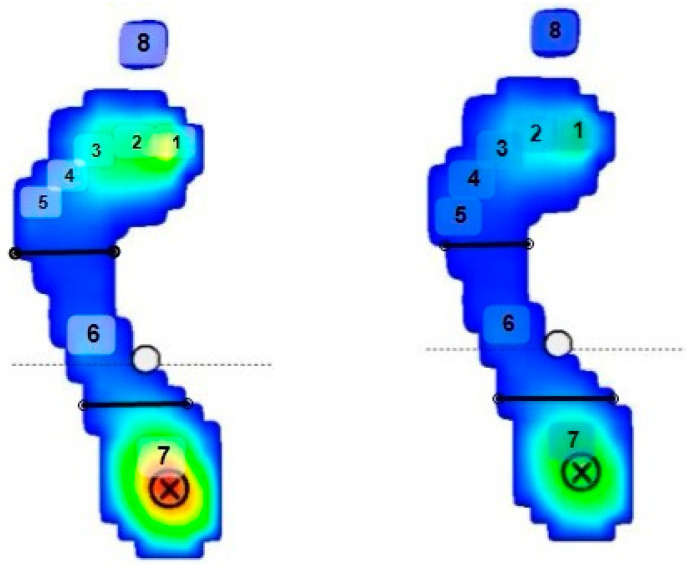
The image shows how the foot was divided into eight regions to obtain the variables. (1) First metatarsal; (2) second metatarsal; (3) third metatarsal; (4) fourth metatarsal; (5) fifth metatarsal; (6) midfoot; (7) hindfoot; and (8) hallux. The circle with the cross indicates the area of the greatest pressure of the plantar footprint. From lowest to May, the pressures are coloured in dark blue, light blue, green, yellow, and the maximum pressure is coloured in red.

For pressure analysis, the foot regions were segmented into distinct areas. The forefoot was transversely subdivided into six regions corresponding to the first, second, third, fourth, and fifth metatarsal heads, and the hallux. Additionally, the midfoot and rearfoot were considered (Figure 1). Subsequently, measurements were taken for the maximum, average, and minimum pressures exerted on each region. Furthermore, the static foot pressure distribution ratio was calculated.

### 2.5. Statistical Analysis

All the data underwent normality testing utilising the Kolmogorov–Smirnov test. The data are deemed to follow a normal distribution when *p* > 0.05. Descriptive statistical analysis presented results as mean ± SD and a 95% confidence interval. To compare demographic variables between male and female participants, an independent *t*-test was employed. Additionally, paired *t*-tests were executed to evaluate potential systematic differences in gait parameters.

For each intrasession test, the intraclass correlation coefficient (ICC) was evaluated using the classification proposed by Landis and Koch. ICC values ranging between 0.20 and 0.40 were deemed to indicate reasonable reliability. Scores falling between 0.40 and 0.60 denoted moderate reliability, while those in the range of 0.60 to 0.80 represented considerable reliability. The highest category encompassed scores between 0.80 and 1.00, characterised as nearly perfect. Some authors suggest that an ICC value of at least 0.75 is necessary to establish reliability [28]. According to the recommendations of Portney and Watkins, clinical measurements with reliability coefficients surpassing 0.90 enhance the likelihood of the measurement being valid [29].

Coefficients of variation (CV) were computed to compare parameters directly in absolute terms. The CV was utilised to describe the connection between the mean magnitude and each studied variable’s variability.

The standard error of measurement (SEM) was used for each variable studied, and, for its best interpretation, it was expressed as a percentage of the mean (SEM%), (138) as follows: SEM is derived from the ICC and DS: SEM = DS × sqrt (1 − ICC), and SEM % = SEM/mean × 100%.

The minimum detectable change (MDC) was also determined. MDC refers to the extent of variation in the value of each scale below which a change can be interpreted as originating from the inherent variability of the assessment method itself, rather than an actual alteration in the patient’s clinical condition. The MDC was computed using a standardised mean (95% MDC), (139), following this formula: MDC is derived from SEM, where MDC = 1.96 × SEM × sqrt (MDC%). Statistical significance was established for *p*-values < 0.05. Finally, normality values were defined for the sample under study, encompassing all the variables obtained. These values were derived from VN = Mean ± 1.96 × SD.

All statistical analyses were conducted using SPSS 17.0 (SPSS Inc., Chicago, IL, USA). The measurement data collected before and after the implementation of the Morton’s extension were processed, considering both static and dynamic aspects within a repeated-measures design. Statistical significance was established at a *p* value of <0.05, and a confidence interval of 95% was utilised.

## 3. Results

The study included a total of 25 participants, comprising 9 men and 16 women. The participants’ ages ranged from 18 to 60 years, with a mean age of 40.36 years and a standard deviation of 15.41 years. The participants had a mean body mass index (BMI) of 24.67 kg/m², with a standard deviation of 5.59 kg/m². Comparing between men and women, there were no significant differences in age and BMI. However, significant differences were observed in weight and height between the two groups. A detailed overview of the participants’ demographic and anthropometric characteristics is displayed in Table 1.

Table 2 shows the results of the intrasession reliability analysis for the right foot and left foot of the variables studied in static and dynamic modes without a Morton’s extension and with a Morton’s extension. The results show a statistically significant relationship, better in static variables than in dynamic variables.

Of the 64 variables studied in statics with and without the Morton’s extension, 58 have an ICC more than 0.80. Significantly, it can be observed in the variables Static. P. Max. 1.meta; Static. P. Max. 2 meta; Static. P. Max. 3 meta; Static. P. Max. 1. meta. MEX; Static. P. Max. 2 meta. MEX ; and Static. P. Max. 3 meta. MEX in both feet. In dynamics, of the 72 variables studied with the Morton’s extension and without the Morton’s extension, 57 have an ICC more than 0.80. Significantly, it can be observed in the right foot variables Dynamic. P. Max. 1. meta; Dynamic. P .Max. 2 .meta; Dynamic. P .Max. 2. meta. MEX; and Dynamic. P. Max. 3. Meta .MEX, and in the left foot variables Dynamic. P. Max. 1 .meta and Dynamic. P. Max. 2. meta. It could be seen in the ANEXO.

**Scheme 1:**
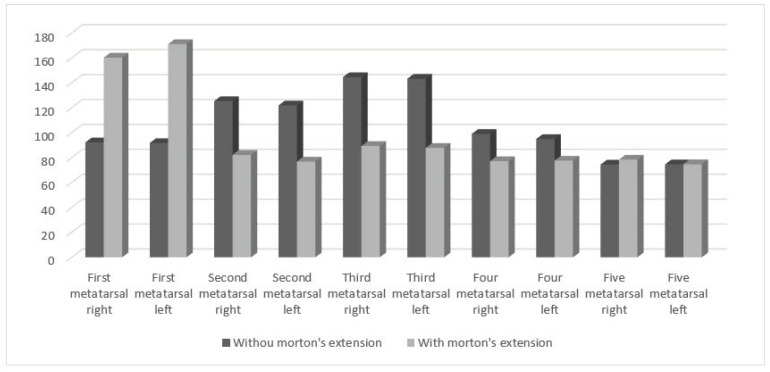
Differences in the maximum pressures obtained in the five metatarsal heads for the right foot and left foot without a Morton’s extension and with a Morton’s extension in static mode.

Scheme 1 shows the comparative histogram of the maximum plantar pressures obtained at each metatarsal head. The light grey colour represents the plantar pressures with a Morton’s extension and dark grey without a Morton’s extension. In this variable, the second-to-fourth metatarsal heads in both feet are lower with a Morton’s extension. However, in the first metatarsal head, the maximum pressure without a Morton’s extension is higher, with a very observable difference graphically, much more than in the rest of the variables. Significantly, it can be observed, a significant reduction in plantar pressures (*p* < 0.05) in the head of the second metatarsal right, with a mean of 125.35 ± 44.48 with a Morton’s extension and 82.24 ± 36.94 without a Morton’s extension, in the head of the second metatarsal left, with a mean of 121.92 ± 46.03 with a Morton’s extension and 76.87 ± 32.69 without a Morton’s extension, in the head of the third metatarsal right, with a mean of 144.62 ± 49.41 with a Morton’s extension and 89.36 ± 44.47 without a Morton’s extension, and in the head of the third metatarsal left, with a mean of 143.35 ± 50.58 with a Morton’s extension and 87.88 ± 43.91 without a Morton’s extension.

**Scheme 2:**
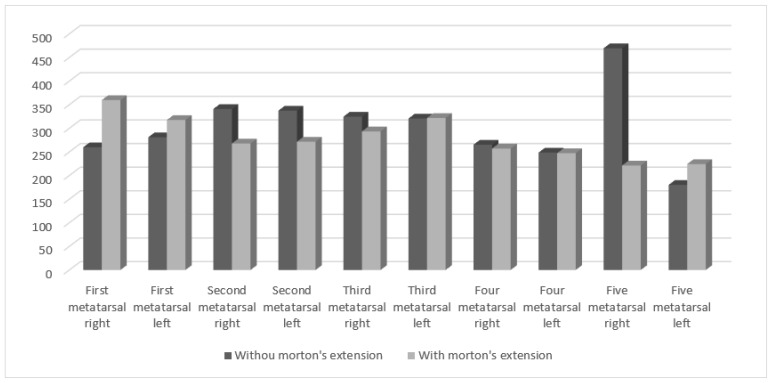
Differences in the maximum pressures obtained in the five metatarsal heads without a Morton’s extension and with a Morton’s extension in dynamic.

Scheme 2 shows the comparative histogram of the maximum plantar pressures obtained at each metatarsal head. The light grey colour represents the plantar pressures with a Morton’s extension and dark grey without a Morton’s extension. In this variable, it can be seen that pressures in the right and left head of the second metatarsal and the right head of the third metatarsal are lower with a Morton’s extension. However, in the first metatarsal head the maximum pressure without Morton’s extension is higher. Significantly, it can be observed, a significant reduction in plantar pressures (*p* < 0.05) in the head of the second metatarsal right, with a mean of 341.05 ± 33.65 with a Morton’s extension and 267.90 ± 44.72 without a Morton’s extension, in the head of the second metatarsal left, with a mean of 337.49 ± 34.18 with a Morton’s extension and 271.64 ± 26.32 without a Morton’s extension, and in the head of the third metatarsal right, with a mean of 324.76 ± 42.32 with a Morton’s extension and 293.53 ± 52.59 without a Morton’s extension.

Table 3 shows the results of the statistical tests for the means obtained in statics of the right foot and left foot with a Morton’s extension and without a Morton’s extension, which show a significant reduction in plantar pressures (*p* < 0.05) in 15 of total variables. The most significant variables are: Static. P. Max. 2 meta. Right (*p* < 0.01); Static. P. Max. 2 meta. Left (*p* < 0.01); Static. P. Max. 3 meta. D (*p* < 0.01); Static. P. Max. 3 meta. Left (*p* < 0.01); Static. P. Max. 4 meta. Right (*p* < 0.01); Static. P. Max. 4 meta. Left (*p* < 0.01); Static. P. Average pressure. 2 meta. Right (*p* < 0.01); Static. P. Average pressure. 2 meta. Left (*p* < 0.01); Static. P. Average pressure. 3 meta. Right (*p* < 0.01); and Static. P. Average pressure. 3 meta Left (*p* < 0.01).

**Figure 2:**
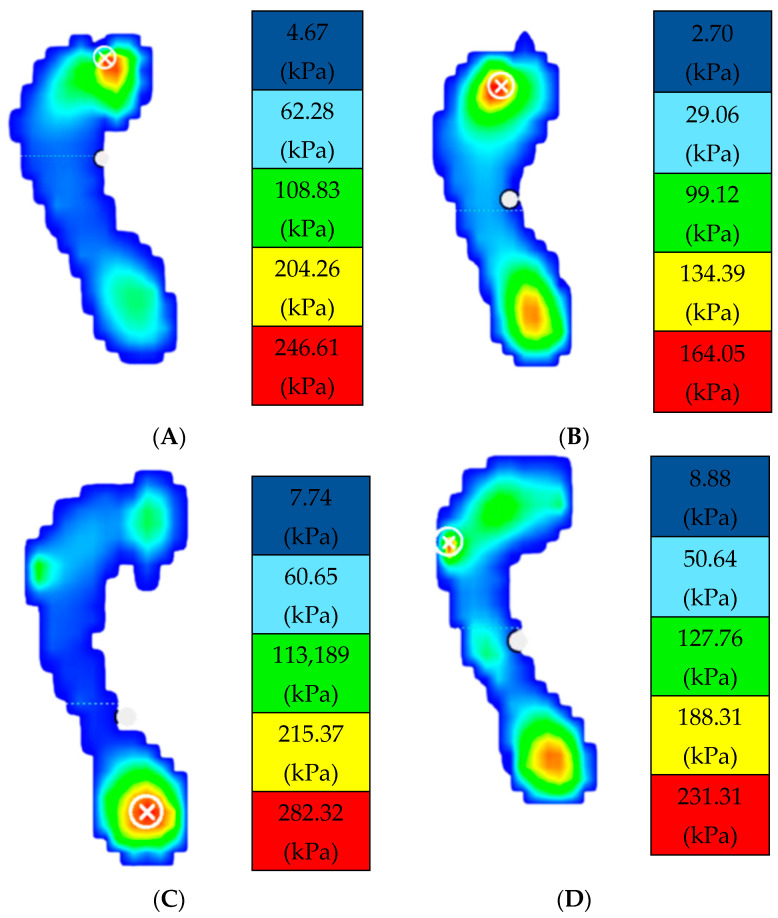
Image (**A**,**B**) shows the static plantar pressures obtained in a woman with a Morton’s extension and without a Morton’s extension. Image (**C**,**D**) shows the plantar pressures obtained in a man with a Morton’s extension and without a Morton’s extension. The circle with the cross indicates the area of the greatest pressure of the plantar footprint. From lowest to May, the pressures are coloured in dark blue, light blue, green, yellow, and the maximum pressure is coloured in red.

A representative image of a plantar pressures platform of one participant before and after orthopaedic simulation is shown in Figure 2.

Table 4 shows the result of the dynamic test for the means obtained in statics of the right foot and left foot with a Morton’s extension and without a Morton’s extension.

The results show a significant reduction in plantar pressures (*p* < 0.05) in 17 of 34 variables, most of them in the first three metatarsals:

Dynamic. P. Max. 1 meta Right (*p* < 0.01); Dynamic. P. Max. 1 meta Left (*p* = 0.003); Dynamic. P Max. 2 meta Right (*p* < 0.01); Dynamic. P. Max. 2 meta Left (*p* < 0.01); Dynamic. P. Max. 3. Meta Right (*p* = 0.005); Dynamic.P.Max.3.meta.Left(*p* = 0.002); Dynamic P Average pressure.1.meta.Right (*p* < 0.01); Dynamic. P. Average pressure 1 meta Left (*p* < 0.01); Dynamic P. Average pressure.2.meta.Right(*p* < 0.01); Dynamic. P. Average pressure.2.meta.Left (*p* < 0.01); Dynamic. P. Average pressure .3. meta Right (*p* < 0.01); Dynamic. P. Average pressure.3.meta. Left (*p* = 0.001); Dynamic. P. Average pressure. 5 meta Left (*p* = 0.002); Dynamic. P. Max. Rft Left (*p* = 0.006); Dynamic. P. Max. Hallux Left (*p* = 0.002); Dynamic. P. Average pressure Hallux Right (*p* = 0.009); and Dynamic. P. Average pressure Hallux Left (*p* = 0.004).

## 4. Discussion

This study aimed to analyse the impact of the Morton’s extension application on variations in plantar pressures in the forefoot. The main goal was to ascertain if applying a 5 mm Morton’s extension could effectively decrease pressures on the lesser metatarsals. The key finding revealed that the Morton’s extension reduced peak pressure in the forefoot compared with the control condition.

The central finding of this study is that the Morton’s extension exerts a direct influence on forefoot pressures, particularly in the hallux, first, second, and third metatarsals, compared with control pressures (without a Morton’s extension). Our hypothesis is corroborated by previous research, where diverse therapeutic strategies such as applying a metatarsal bar while walking [27] and running [8], along with orthoses designed with a medial arch support [30], were shown to intervene directly in reducing the pressures under investigation. The Morton’s extension diminishes the pressure beneath the second and third metatarsal heads by redistributing the pressure load to neighbouring tissues. This phenomenon potentially enhances sensory input from deep pressure receptors (large-fibre afferents) in the adjacent tissue, suggesting a possible modulation of metatarsalgia according to the gate-control theory [31,32]. A study by Hodge et al. determined that only 32% of pain variance in metatarsalgia was attributed to average pressure beneath the metatarsal head.

Consequently, it was hypothesised that pain sensation might contribute to this observation [33]. Similarly, Postema et al. observed that subjects experiencing pain preferred custom-moulded insoles [34], which alleviated metatarsal head loading and harmonised plantar pressures [12,35]. Thus, insoles may mitigate forefoot issues [36]. We identified an increase in pressure at the head of the first metatarsal, both in static and dynamic conditions. Plantar pressures arise from the vertical force applied during walking, divided by the contact area. This assumes the sensors’ spatial resolution was sufficient and that the plantar surface maintained full contact with the measurement platform [36]. Morton’s various studies describe a connection between hypermobility of the first tarsometatarsal joint (TMTJ1) and foot disorders [37]. Consequently, the adjacent metatarsals and the longitudinal arch are affected due to this instability in the first ray. Therefore, we posit that stabilising the first metatarsophalangeal joint directly influences plantar pressures on the lesser metatarsals, reducing them.

In the existing literature, no prior evidence exists regarding using the Morton’s extension to assess its impact on variations in plantar pressures. Consequently, comparing the outcomes of our study with those of other researchers proves challenging. Nonetheless, our methodology stands as a significant strength.

The Morton’s extension material chosen for this study is among the most commonly employed in the design of plantar orthoses [38,39,40,41]. The measurements conducted have been repeated extensively to ensure the reliability of the analytical data. Specifically, each of the 25 study participants underwent 24 analyses. The selection algorithm for the eight designated areas for plantar pressure analysis was determined based on prior research [40]. Although previous studies did not subdivide the five metatarsal heads [41], Deschamps et al., in a reliability study concerning plantar pressure measurements, concluded that, while precision is imperative, reliability improves when conducted by a single observer [42]. In line with the findings of Van der Leeden et al., an average of three readings was also chosen for the present study, as they demonstrated that a minimum of three measurements yielded a consistent average [5].

Our findings highlight variations dependent on the specific foot under analysis. These outcomes align with those discovered by Yoon et al., who proposed significant disparities in forefoot pressure between the left and right feet when comparing pressures before and after the placement of a metatarsal bar [27]. This observation prompts us to treat the data distinctly for each foot. Another question that piqued our interest was whether the use of the EXM induced pressure variability in the midfoot and rearfoot. Earlier investigations indicate that employing a metatarsal bar increases the contact area of the forefoot [41,43]. These results correspond with findings in other studies where weight distribution is believed to reduce plantar pressures across the entire contact area [44]. In contrast to our results, where the application of the EXM did not yield significant changes in the contact area, we speculate that pressures in the midfoot and rearfoot remained unchanged.

Our findings have several implications; utilising real-time plantar pressure measurements before and after placing the Morton’s extension can be a valuable tool for monitoring treatment in clinical settings. Nonetheless, this study does possess some limitations. The Morton’s extension remained consistent in material, thickness, and hardness throughout the study. Body mass index was considered, as the sample primarily consisted of individuals categorised as normal weight or overweight, akin to other studies [21,45,46]. Nevertheless, further investigations are warranted, especially encompassing obesity, as effects may diverge. Similarly, studies differentiated by gender are also necessary, given the potential differences between men and women. Additionally, it is worth noting that the observed effects on plantar pressures could be better understood over time through a longitudinal study design. This study effectively accomplishes its research objectives by highlighting the immediate impacts of the orthopaedic Morton’s extension application. However, forthcoming research is imperative to validate any potential long-term effects.

## 5. Conclusions

In summary, the Morton’s extension induces alterations in plantar pressures. Specifically, in static conditions, it leads to heightened pressure in the first metatarsal and the hallux. Conversely, a noteworthy reduction in pressure is observed in the second, third, and fourth metatarsals. Notably, the fifth metatarsal, midfoot, and rearfoot remain unaffected.

In the dynamic aspect, we also observe elevated pressure in the first metatarsal and the hallux. Notably, there is a marked decrease in pressure across the second and third metatarsals. Conversely, pressures in the fourth and fifth metatarsals, and the midfoot and rearfoot remain unaltered, demonstrating no significant changes.

We propose that the application of the Morton’s extension for individuals with metatarsalgia should be tailored and fine-tuned based on ongoing monitoring of plantar pressure measurements.

Tailored insoles incorporating the Morton’s extension can potentially mitigate plantar pressure on the second and third metatarsal bones. By reducing metatarsal head loading and the balanced redistribution of plantar pressures, such insoles may effectively alleviate metatarsalgia within these metatarsal regions, both in static and dynamic conditions.

Within clinical practice, numerous patients express concerns about pain or deformities in the forefoot. Introducing adjustments to footwear and implementing insoles or orthoses can offer substantial benefits in addressing such forefoot issues. This study serves as a valuable resource for clinicians, equipping them to explore a range of strategies for managing forefoot disorders.

## Figures and Tables

**Table 1:** Demographics data for total population and gender.

Descriptive Data	Male (*n* = 9) Mean ± SD (95%CI)	Female (*n* = 16) Mean ± SD (95%CI)	Total (*n* = 25) Mean ± SD (95%CI)	*p*-Value
Age (years)	38.11 ± 15.96 (25.84–50.38)	41.63 ± 15.47 (33.38–49.87)	40.36 ± 15.41 (34.00–46.72)	0.595
Height (cm)	178.89 ± 4.31 (157.57–182.20)	162.94 ± 5.43 (160.04–165.83)	168.68 ± 9.25 (164.86–172.50)	<0.001
Weight (kg)	88.11 ± 15.43 (76.25–99.97)	61.63 ± 12.67 (54.87–68.38)	71.16 ± 18.65 (63.46–78.86)	<0.001
BMI (Kg/m^2^)	27.52 ± 4.56 (24.01–31.03)	23.07 ± 5.59 (20.09–26.05)	24.67 ± 5.59 (22.36–26.98)	0.540

Abbreviations: BMI, body mass index; Kg, kilograms; CM, centimetres; SD, standard deviation; CI, confidence interval. The student’s *t*-test for independent samples was applied. In all analyses, *p* < 0.05 (with a 95% confidence interval) was considered statistically significant.

**Table 2:** Analysis of the intrasession reliability of the variables studied in static and dynamic modes without a Morton’s extension and with a Morton’s extension for the right foot and left foot, and normality values in the total population.

VARIABLE (*n* = 68)	Right Foot			Left Foot		
	ICC (95% CI)	SEM	VN ± SD (VN Inferior-VN Superior)	ICC (95% CI)	SEM	VN ± SD (VN Inferior-VN Superior)
Static. P. Max. 1 meta. (kPa)	0.953 (0.908–0.978)	9.73	92.24 ± 87.96 (4.28; 180.20)	0.859 (0.726–0.933)	16.28	91.74 ± 84.97 (6.77; 176.71)
Static. P. Max. 2 meta. (kPa)	0.905 (0.813–0.955)	13.71	125.35 ± 87.18 (38.17; 212.53)	0.900 (0.806–0.953)	14.56	121.92 ± 90.22 (31.70; 212.14)
Static. P. Max. 3 meta. (kPa)	0.923 (0.850–0.963)	13.71	144.62 ± 96.84 (47.78; 241.46)	0.928 (0.860–0.966)	13.49	143.35 ± 98.55 (44.80; 241.90)
Static. P. Max. 4 meta. (kPa)	0.926 (0.857–0.965)	11.06	99.04 ± 79.65 (19.39; 178.69)	0.899 (0.804–0.952)	14.58	94.92 ± 89.94 (4.98; 184.86)
Static. P. Max. 5 meta. (kPa)	0.961 (0.925–0.982)	10.63	74.44 ± 105.51 (−31.07; 179.95)	0.959 (0.920–0.980)	12.14	74.58 ± 117.48 (−42.90; 192.06)
Static. P. Average pressure. 1 meta. (kPa)	0.966 (0.934–0.984)	6.85	67.13 ± 72.85 (−5.72; 139.98)	0.944 (0.890–0.973)	7.28	66.26 ± 60.29 (5.97; 126.55)
Static. P. Average pressure. 2 meta. (kPa)	0.950 (0.903–0.976)	9.22	105.07 ± 80.85 (24.22; 185.92)	0.913 (0.832–0.959)	11.05	99.02 ± 73.44 (25.58; 172.46)
Static. P. Average pressure. 3 meta. (kPa)	0.945 (0.893–0.974)	11.01	120.11 ± 92.00 (28.11; 212.11)	0.958 (0.918–0.980)	8.96	118.07 ± 85.65 (32.42; 203.72)
Static. P. Average pressure. 4 meta. (kPa)	0.965 (0.932–0.984)	7.17	77.12 ± 75.09 (2.03; 152.21)	0.964 (0.930–0.983)	6.61	71.14 ± 68.31 (2.83; 139.45)
Static. P. Average pressure. 5 meta. (kPa)	0.988 (0.977–0.994)	5.03	52.05 ± 90.00 (−37.95; 142.05)	0.981 (0.963–0.991)	6.78	50.27 ± 96.47 (−46.20; 146.74)
Static. P. Max. Mdf. (kPa)	0.953 (0.908–0.978)	3.31	16.67 ± 29.97 (−13.30; 46.64)	0.981 (0.962–0.991)	3.73	20.12 ± 53.00 (−32.88; 73.12)
Static. P. Average pressure. Mdf. (kPa)	0.974 (0.949–0.988)	2.04	10.40 ± 24.75 (−14.35; 35.15)	0.955 (0.912–0.979)	3.47	11.95 ± 32.07 (−20.12; 44.02)
Static. P. Max. Rft. (kPa)	0.794 (0.600–0.903)	16.83	223.52 ± 72.66 (150.86; 296.18)	0.792 (0.595–0.902)	18.53	253.22 ± 79.62 (173.60; 332.84)
Static. P. Average pressure. Rft. (kPa)	0.891 (0.789–0.949)	12.49	198.34 ± 74.17 (124.17; 272.51)	0.953 (0.909–0.978)	10.44	216.65 ± 94.37 (122.28; 311.02)
Static. P. Max. Hallux. (kPa)	0.002 (−0.941–0.528)	4930.97	1014.28 ± 9674.38 (−8660.10; 10,688.66)	0.594 (0.210–0.808)	15.52	19.92 ± 47.75 (−27.83; 67.67)
Static. P. Average pressure. Hallux. (kPa)	0.951 (0.905–0.77)	3.69	11.50 ± 32.71 (−21.21; 44.21)	0.872 (0.751–0.939)	2.50	6.21 ± 13.68 (−7.47; 19.89)
Dynamic. P. Max. 1 meta. (kPa)	0.944 (0.890–0.973)	18.18	259.96 ± 150.55 (109.41; 410.51)	0.877 (0.761–0.942)	21.14	281.16 ± 118.13 (163.03; 399.29)
Dynamic. P. Max. 2 meta. (kPa)	0.833 (0.676–0.921)	13.75	341.05 ± 65.95 (275.10; 407.00)	0.896 (0.798–0.951)	11.02	337.49 ± 66.99 (270.50; 404.48)
Dynamic. P. Max. 3 meta. (kPa)	0.458 (−0.54–0.743)	31.16	324.76 ± 82.95 (241.81; 407.71)	0.687 (0.391–0.852)	24.62	320.77 ± 86.24 (234.53; 407.01)
Dynamic. P. Max. 4 meta. (kPa)	0.945 (0.893–0.974)	13.41	265.42 ± 112.03 (153.39; 377.45)	0.956 (0.914–0.979)	13.16	248.66 ± 122.97 (125.69; 371.63)
Dynamic P. Max. 5 meta. (kPa)	−0.001 (−0.945–0.526)	1306.54	469.54 ± 2559.54 (−2090.00; 3029.08)	0.923 (0.850–0.936)	25.04	180.22 ± 176.87 (3.35; 357.09)
Dynamic. P. Average pressure. 1 meta. (kPa)	0.923 (0.851–0.964)	13.33	109.06 ± 94.18 (14.88; 203.24)	0.796 (0.603–0.903)	15.99	125.59 ± 69.40 (56.19; 194.99)
Dynamic. P.Average pressure. 2 meta. (kPa)	0.924 (0.853–0.964)	9.06	171.06 ± 64.44 (106.62; 235.50)	0.935 (0.874–0.969)	7.79	167.51 ± 59.88 (107.63; 227.39)
Dynamic. P.Average pressure. 3 meta. (kPa)	0.948 (0.898–0.975)	11.15	171.64 ± 95.82 (75.82; 267.46)	0.976 (0.952–0.989)	6.91	158.67 ± 87.38 (71.29; 246.05)
Dynamic. P.Average pressure. 4 meta. (kPa)	0.946 (0.895–0.975)	10.36	119.52 ± 87.42 (32.10; 206.94)	0.947 (0.897–0.975)	10.51	104.7 ± 89.45 (15.25; 194.15)
Dynamic. P.Average pressure. 5 meta. (kPa)	0.922 (0.848–0.963)	13.32	79.6 ± 93.49 (−13.89; 173.09)	0.907 (0.818–0.956)	13.32	62.16 ± 85.59 (−23.43; 147.75)
Dynamic. P. Max. Mdf. (kPa)	0.933 (0.869–0.968)	9.76	34.48 ± 73.93 (−39.45; 108.41)	0.947 (0.896–0.975)	9.90	32.65 ± 84.26 (−51.61; 116.91)
Dynamic. P.Average pressure. Mdf. (kPa)	0.938 (0.879–0.971)	2.86	9.97 ± 22.50 (−12.53; 32.47)	0.935 (0.874–0.969)	3.55	9.87 ± 27.30 (−17.43; 37.17)
Dynamic. P. Max. Rft. (kPa)	0.772 (0.558–0.892)	12.51	321.97 ± 51.33 (270.64; 373.30)	0.911 (0.826–0.958)	10.11	340.37 ± 66.44 (273.93; 406.81)
Dynamic. P.Average pressure. Rft. (kPa)	0.951 (0.904–0.977)	8.09	132.77 ± 71.62 (61.15; 204.39)	0.832 (0.673–0.920)	11.00	140.39 ± 52.61 (87.78; 193.00)
Dynamic. Contact.surfaces. (kPa)	0.984 (0.969–0.992)	4.24	106.77 ± 65.70 (41.07; 172.47)	0.948 (0.900–0.976)	7.09	101.69 ± 60.94 (40.75; 162.63)
Dynamic. Step.duration. (kPa)	0.930 (0.865–0.967)	32.54	767.18 ± 241.08 (526.10; 1008.26)	0.921 (0.847–0.963)	36.13	764.65 ± 251.98 (512.67; 1016.63)
Dynamic. P. Max. Hallux. (kPa)	0.837 (0.682–0.923)	28.69	242.50 ± 139.30 (103.20; 381.80)	0.752 (0.519–0.883)	33.94	264.62 ± 133.57 (131.05; 398.19)
Dynamic. P. Average pressure. Hallux. (kPa)	0.901 (0.808–0.953)	12.67	78.14 ± 78.95 (−0.81; 157.09)	0.726 (0.468–0.870)	19.03	83.85 ± 71.25 (12.60; 155.10)
Static. P. Max.1 meta. MEX (kPa)	0.936 (0.876–0.970)	14.26	160.24 ± 110.49 (49.75; 270.73)	0.935 (0.873–0.969)	15.06	171.11 ± 115.78 (55.33; 286.89)
Static. P. Max. 2 meta. MEX (kPa)	0.972 (0.945–0.987)	6.18	82.24 ± 72.40 (9.84; 154.64)	0.956 (0.915–0.979)	6.86	76.87 ± 64.07 (12.80; 140.94)
Static. P. Max. 3 meta. MEX (kPa)	0.973 (0.948–0.987)	7.36	89.36 ± 87.75 (1.61; 177.11)	0.971 (0.944–0.986)	7.48	87.88 ± 86.06 (1.82; 173.94)
Static. P. Max. 4 meta. MEX (kPa)	0.976 (0.953–0.989)	6.10	77.20 ± 77.13 (0.07; 154.33)	0.985 (0.972–0.993)	5.62	77.60 ± 89.91 (−12.31; 167.51)
Static. P. Max. 5 meta. D.MEX (kPa)	0.940 (0.883–0.972)	12.01	78.41 ± 96.10 (−17.69; 174.51)	0.988 (0.976–0.994)	6.07	74.69 ± 108.62 (−33.93; 183.31).
Static. P. Average pressure. 1 meta. MEX (kPa)	0.945 (0.892–0.974)	12.99	123.05 ± 108.60 (14.45; 231.65)	0.847 (0.703–0.928)	19.75	130.64 ± 98.98 (31.66; 229.62)
Static. P. Average pressure. 2 meta. MEX (kPa)	0.971 (0.944–0.986)	6.00	67.39 ± 69.09 (−1.70; 136.48)	0.983 (0.967–0.992)	3.89	60.36 ± 58.51 (1.85; 118.87)
Static. P. Average pressure. 3 meta. MEX (kPa)	0.973 (0.947–0.987)	7.10	75.86 ± 84.71 (−8.85; 160.57)	0.976 (0.954–0.989)	6.35	72.70 ± 80.38 (−7.68; 153.08)
Static. P. Average pressure. 4 meta. MEX (kPa)	0.965 (0.933–0.984)	6.82	63.59 ± 71.50 (−7.91; 135.09)	0.984 (0.969–0.992)	5.01	60.94 ± 77.56 (−16.62; 138.50)
Static. P. Average pressure. 5 meta. MEX (kPa)	0.955 (0.912–0.979)	9.03	56.68 ± 83.48 (−26.80; 140.16)	0.987 (0.974–0.994)	4.96	50.24 ± 85.22 (−34.98; 135.46)
Static. P. Max. Mdf. MEX (kPa)	0.979 (0.960–0.990)	2.32	17.57 ± 31.40 (−13.83; 48.97)	0.970 (0.942–0.986)	3.50	16.19 ± 39.65 (−23.46; 55.84)
Static. P. Average pressure. Mdf. MEX (kPa)	0.982 (0.965–0.991)	1.79	11.49 ± 26.19 (−14.70; 37.68)	0.978 (0.958–0.990)	2.38	11.36 ± 31.44 (−20.08; 42.80)
Static. P. Max. Rft. MEX (kPa)	0.888 (0.783–0.947)	16.64	213.47 ± 97.45 (116.02; 310.92)	0.931 (0.867–0.968)	14.12	236.68 ± 105.35 (131.33; 342.03)
Static. P. Average pressure. Rft. MEX (kPa)	0.967 (0.935–0.984)	8.26	194.16 ± 89.08 (105.08; 283.24).	0.969 (0.939–0.985)	9.13	212.6 ± 101.59 (111.01; 314.19)
Static. P. Max. Hallux. MEX (kPa)	0.910 (0.826–0.958)	13.24	24.19 ± 86.49 (−62.30; 110.68).	0.921 (0.847–0.963)	5.92	12.16 ± 41.26 (−29.10; 53.42)
Static. P. Average pressure. Hallux. MEX (kPa)	0.931 (0.866–0.967)	7.67	12.09 ± 57.23 (−45.14; 69.32)	0.919 (0.842–0.961)	4.13	6.32 ± 28.46 (−22.14; 34.78)
Dynamic. P. Max. 1 meta. MEX (kPa)	0.300 (−0.886–0.541)	117.61	359.86 ± 275.52 (84.34; 635.38)	0.718 (0.451–0.866)	22.16	317.63 ± 81.79 (235.84; 399.42)
Dynamic. P. Max. 2 meta. MEX (kPa)	0.930 (0.86–0.967)	11.83	267.90 ± 87.65 (180.25; 355.55)	0.643 (0.306–0.831)	15.73	271.64 ± 51.59 (220.05; 323.23)
Dynamic. P. Max. 3 meta. MEX (kPa)	0.897 (0.800–0.951)	16.88	293.53 ± 103.08 (190.45; 396.61)	0.051 (−0.845–0.551)	153.35	321.94 ± 308.54 (13.40; 630.48)
Dynamic. P. Max. 4 meta. MEX (kPa)	0.949 (0.902–0.976)	14.16	257.20 ± 122.89 (134.31; 380.09)	0.930 (0.865–0.967)	18.94	247.45 ± 140.34 (107.11; 387.79)
Dynamic P. Max. 5 meta. MEX (kPa)	0.921 (0.846–0.962)	24.61	221.32 ± 171.60 (49.72; 392.92).	0.924 (0.853–0.964)	27.31	224.41 ± 194.18 (30.23; 418.59)
Dynamic. P. Average pressure. 1 meta.MEX (kPa)	0.786 (0.585–0.899)	16.47	165.39 ± 69.78 (95.61; 235.17)	0.808 (0.626–0.909)	13.31	170.81 ± 59.53 (111.28; 230.34).
Dynamic. P.Average pressure. 2 meta.MEX (kPa)	0.954 (0.910–0.978)	8.25	129.35 ± 75.36 (53.99; 204.71)	0.872 (0.751–0.939)	9.60	130.84 ± 52.57 (78.27; 183.41)
Dynamic. P.Average pressure. 3 meta.MEX (kPa)	0.956 (0.914–0.979)	10.38	140.47 ± 97.00 (43.47; 237.47).	0.930 (0.865–0.967)	11.21	138.41 ± 83.05 (55.36; 221.46)
Dynamic. P.Average pressure. 4 meta.MEX (kPa)	0.968 (0.938–0.985)	9.96	118.91 ± 109.13 (9.78; 228.04)	0.024 (−0.896–0.538)	1244.66	265.68 ± 2469.35 (−2203.67; 2735.03).
Dynamic. P.Average pressure. 5 meta.MEX (kPa)	0.894 (0.794–0.950)	17.18	88.13 ± 103.43 (−15.30; 191.56)	0.969 (0.940–0.985)	10.56	89 ± 117.52 (−28.52; 206.52)
Dynamic. P. Max. Mdf.MEX (kPa)	0.934 (0.872–0.969)	11.93	40.1 ± 91.02 (−50.92; 131.12).	0.935 (0.875–0.969)	11.45	35.65 ± 88.00 (−52.35; 123.65).
Dynamic. P.Average pressure. Mdf.MEX (kPa)	0.880 (0.767–0.943)	4.50	11.12 ± 25.48 (−14.36; 6.60).	0.935 (0.874–0.969)	3.44	10.09 ± 26.44 (−16.35; 36.53)
Dynamic. P. Max. Rft.MEX (kPa)	0.425 (−0.118–0.728)	24.80	317.95 ± 64.09 (253.86; 382.04)	0.692 (0.400–0.854)	20.90	323.7 ± 73.81 (249.89; 397.51)
Dynamic. P. Average pressure.Rft.MEX (kPa)	0.915 (0.834–0.960)	10.04	135.57 ± 67.48 (68.09; 203.05)	0.925 (0.855–0.965)	9.85	131.62 ± 70.52 (61.10; 202.14)
Dynamic. Contact.surfaces.MEX (kPa)	0.989 (0.979–0.995)	2.53	103.61 ± 47.29 (56.32; 150.90)	0.882 (0.771–0.944)	8.46	100.60 ± 48.27 (52.33; 148.87)
Dynamic. Step.duration.MEX (kPa)	0.956 (0.915–0.979)	23.52	755.59 ± 219.79 (535.80; 975.38)	0.954 (0.911–0.978)	25.54	762.61 ± 233.38 (529.23; 95.99)
Dynamic. P. Max. Hallux. MEX (kPa)	0.935 (0.873–0.969)	24.11	219.47 ± 185.38 (34.09; 404.85)	0.914 (0.832–0.959)	21.86	212.44 ± 146.12 (66.32; 358.56)
Dynamic. P. Average pressure. Hallux. MEX (kPa)	0.893 (0.792–0.949)	13.22	62.81 ± 79.18 (−16.37; 141.99)	0.868 (0.746–0.937)	12.95	58.92 ± 69.83 (−10.91; 128.75).

Abbreviations: P, pressure; Max, maximum; 1 meta, first metatarsal; 2 meta, second metatarsal; 3 meta, third metatarsal; 4 meta, fourth metatarsal; 5 meta, fifth metatarsal; Mdf, midfoot; Rft, rearfoot; Kpa, kilopascals; SD, standard deviation; CV, coefficients of variation; ICC, intraclass correlation coefficient; CI, confidence interval; SEM, standard error of the mean; MDC, minimum detectable change; VN, normal values. *p* < 0.05 (with a 95% confidence interval) was considered statistically significant.

**Table 3:** Differences between the variables for the right foot and left foot with a Morton’s extension and without a Morton’s extension in statics.

VARIABLE	Without Morton’s Extension Average Pressure ± SD (IC95%), *n* = 32	With Morton’s Extension Average Pressure ± SD (IC95%), *n* = 32	*p*-Value	D de Cohen
Static. P.Max. 1 meta.Right (kPa)	92.24 ± 44.88 (73.71–110.76)	160.24 ± 56.37 (136.97–183.51)	<0.01 ^b^	0.55
Static. P. Max. 1 meta. Left (kPa)	91.74 ± 43.35 (73.84–109.64)	171.11 ± 59.07 (146.72–195.49)	<0.01 ^b^	0.60
Static. P. Max. 2 meta.Right (kPa)	125.35 ± 44.48 (106.99–43.72)	82.24 ± 36.94 (66.99–97.49)	<0.01 ^b^	0.46
Static. P. Max. 2 meta.Left (kPa)	121.92 ± 46.03 (102.92–40.92)	76.87 ± 32.69 (63.37–90.36)	<0.01 ^b^	0.49
Static. P. Max. 3 meta.Right (kPa)	144.62 ± 49.41 (124.23–65.02)	89.36 ± 44.77 (70.88–107.84)	<0.01 ^a^	0.50
Static. P. Max. 3 meta.Left (kPa)	143.35 ± 50.28 (122.60–64.11)	87.88 ± 43.91 (69.75–106.01)	<0.01 ^a^	0.50
Static. P. Max. 4 meta.Right (kPa)	99.04 ± 40.64 (82.26–115.82)	77.20 ± 39.35 (60.96–93.45)	<0.01 ^a^	0.26
Static. P. Max. 4 meta. Left (kPa)	94.92 ± 45.89 (75.98–113.87)	77.6 ± 45.87 (58.66–96.54)	0.01 ^a^	0.18
Static. P. Max. 5 meta.Right (kPa)	74.44 ± 53.83 (52.21–96.66)	78.41 ± 49.03 (58.17–98.65)	0.253 ^a^	0.03
Static. P. Max. 5 meta. Left (kPa)	74.58 ± 59.94 (49.84–99.33)	74.69 ± 55.42 (51.81–97.57)	0.459 ^a^	0.0009
Static. P. Average pressure. 1 meta.Right (kPa)	67.13 ± 37.17 (51.79–82.47)	123.05 ± 55.41 (100.17–145.92)	<0.01 ^a^	0.50
Static. P. Average pressure. 1 meta.Left (kPa)	66.26 ± 30.76 (53.56–78.95)	130.64 ± 50.5 (109.79–151.49)	<0.01 ^a^	0.61
Static. P. Average pressure. 2 meta.Right (kPa)	105.07 ± 41.25 (88.04–122.10)	67.39 ± 35.25 (52.84–81.94)	<0.01 ^a^	0.44
Static. P. Average pressure. 2 meta.Left (kPa)	99.02 ± 37.47 (83.56–114.49)	60.36 ± 29.85 (48.04–72.69)	<0.01 ^b^	0.49
Static. P. Average pressure. 3 meta.Right (kPa)	120.11 ± 46.94 (100.73–39.48)	75.86 ± 43.22 (58.02–93.71)	<0.01 ^a^	0.44
Static. P. Average pressure. 3 meta.Left (kPa)	118.07 ± 43.7 (100.03–36.11)	72.7 ± 41.01 (55.78–89.63)	<0.01 ^a^	0.47
Static. P. Average pressure. 4 meta.Right (kPa)	77.12 ± 38.31 (61.31–92.94)	63.59 ± 36.48 (48.53–78.65)	0.001 ^a^	0.17
Static.P. Average pressure. 4 meta.Left (kPa)	71.14 ± 34.85 (56.75–85.53)	60.94 ± 39.57 (44.60–77.27)	0.058 ^a^	0.13
Static . P. Average pressure. 5 meta.Right (kPa)	52.05 ± 45.92 (33.09–71.01)	56.68 ± 42.59 (39.10–74.26)	0.201 ^a^	0.05
Static. P. Average pressure. 5 meta.Left (kPa)	50.27 ± 49.22 (29.95–70.59)	50.24 ± 43.48 (32.30–68.19)	0.288 ^a^	0.0003
Static. P. Max. Mdf.Right (kPa)	16.67 ± 15.29 (10.36–22.99)	17.57 ± 16.02 (10.95–24.18)	0.681 ^a^	0.02
Static. P. Max. Mdf.Left (kPa)	20.12 ± 27.04 (8.96–31.28)	16.19 ± 20.23 (7.84–24.54)	0.480 ^b^	0.08
Static. P. Average pressure. Mdf.Right (kPa)	10.40 ± 12.63 (5.18–15.61)	11.49 ± 13.36 (5.97–17.01)	0.469 ^b^	0.04
Static. P. Average pressure. Mdf. Left (kPa)	11.95 ± 16.36 (5.19–18.70)	11.36 ± 16.04 (4.74–17.98)	0.538 ^b^	0.01
Static. P. Max. Rft.Right (kPa)	223.52 ± 37.07 (208.22–238.82)	213.47 ± 49.72 (192.95–233.99)	0.166 ^b^	0.11
Static. P. Max. Rft.Left (kPa)	253.22 ± 40.62 (208.22–238.82)	236.68 ± 53.75 (214.49–258.87)	0.035 ^b^	0.17
Static P. Average pressure.Rft.Right (kPa)	198.34 ± 37.84 (326.38–354.37)	194.16 ± 45.45 (175.40–212.92)	0.459 ^b^	0.04
Static. P. Average pressure.Rft Left (kPa)	216.65 ± 48.15 (182.71–213.96)	212.60 ± 51.83 (191.20–233.99)	0.510 ^b^	0.04
Static. P. Max. Hallux.Right (kPa)	1014.28 ± 4935.91 (1023.15–051.73)	24.19 ± 44.13 (5.98–42.41)	0.720 ^a^	0.14
Static. P. Max. Hallux.Left (kPa)	19.92 ± 24.36 (9.86–29.98)	12.16 ± 21.05 (3.47–20.85)	0.110 ^a^	0.16
Static. P. Average pressure. Hallux.Right (kPa)	11.50 ± 16.69 (4.60–18.39)	12.09 ± 29.2 (0.03–24.14)	0.179 ^a^	0.01
Static. P. Average pressure. Hallux.Left (kPa)	6.21 ± 6.98 (3.33–9.10)	6.32 ± 14.52 (0.32–12.32)	0.306 ^a^	0.004

Abbreviations: P, pressure; Max, maximum; 1 meta, first metatarsal; 2 meta, second metatarsal; 3 meta, third metatarsal; 4 meta, fourth metatarsal; 5 meta, fifth metatarsal; Mdf, midfoot; Rft, rearfoot; Kpa, kilopascals; SD, standard deviation. *p* < 0.05 (with a 95% confidence interval) was considered statistically significant; Cohen’s d. ^a^ Wilcoxon signed rank test *p*-value < 0.05 considered statistically significant. ^b^ Paired *t*-test *p*-value considered statistically significant.

**Table 4:** Differences between the variables for the right foot and left foot with a Morton’s extension and without a Morton’s extension in dynamics.

VARIABLE	Without Morton’s Extension Average Pressure ± SD (IC95%), *n* = 36	With Morton’s Extension Average Pressure ± SD (IC95%), *n* = 36	*p*-Value	De Cohen
Dynamic. P. Max. 1 meta.Right (kPa)	259.96 ± 76.81 (228.26–91.67)	359.86 ± 140.57 (301.83–417.89)	<0.01 ^a^	0.40
Dynamic. P. Max. 1 meta.Left (kPa)	281.16 ± 60.27 (256.28–06.04)	317.63 ± 41.73 (300.40–334.85)	0.003 ^b^	0.70
Dynamic. P Max. 2 meta.Right (kPa)	341.05 ± 33.65 (327.166–354.94)	267.90 ± 44.72 (249.44–186.36)	<0.01 ^b^	0.67
Dynamic. P. Max. 2 meta.Left (kPa)	337.49 ± 34.18 (323.38–51.60)	271.64 ± 26.32 (260.78–282.51)	<0.01 ^a^	0.73
Dynamic. P. Max. 3 meta.Right (kPa)	324.76 ± 42.32 (307.32–42.27)	293.53 ± 52.59 (271.82–315.24)	0.005 ^b^	0.31
Dynamic. P. Max. 3 meta.Left (kPa)	320.77 ± 44 (302.60–38.93)	321.94 ± 157.42 (256.95–386.92)	0.002 ^a^	0.005
Dynamic. P. Max. 4 meta.Right (kPa)	265.42 ± 57.16 (241.82–89.01)	257.20 ± 62.7 (231.32–283.08)	0.427 ^b^	0.06
Dynamic. P. Max. 4 meta.Left (kPa)	248.66 ± 62.74 (222.76–74.56)	247.45 ± 71.6 (217.89–277.01)	0.638 ^b^	0.008
Dynamic. P. Max. 5 meta.Right (kPa)	469.54 ± 1305.89 (−69.50–1008.58)	221.32 ± 87.55 (185.18–257.46)	0.397 ^a^	0.13
Dynamic. P. Max. 5 meta.Left (kPa)	180.22 ± 90.24 (142.97–217.47)	224.41 ± 99.07 (183.51–265.30)	0.008 ^b^	0.22
Dynamic. P. Average pressure 1 meta.Right (kPa)	109.06 ± 48.05 (89.22–128.90)	165.39 ± 35.6 (150.82–179.97)	<0.01 ^b^	0.55
Dynamic. P. Average pressure. 1 meta.Left (kPa)	125.59 ± 35.41 (110.96–140.21)	170.81 ± 30.37 (158.27–183.35)	<0.01 ^b^	0.56
Dynamic. P. Average pressure. 2 meta.Right (kPa)	171.06 ± 32.88 (157.48–184.63)	129.35 ± 38.45 (113.47–145.22)	<0.01 ^b^	0.50
Dynamic. P. Average pressure. 2 meta.Left (kPa)	167.51 ± 30.55 (154.90–180.12)	130.84 ± 26.82 (119.76–141.91)	<0.01 ^b^	0.53
Dynamic. P. Average pressure. 3 meta.Right (kPa)	171.64 ± 48.89 (151.45–91.82)	140.47 ± 49.49 (120.04–160.90)	<0.01 ^b^	0.30
Dynamic. P. Average pressure. 3 meta. Left (kPa)	158.67 ± 44.58 (140.27–177.07)	138.41 ± 42.37 (120.92–155.91)	0.001 ^b^	0.22
Dynamic. P. Average pressure. 4 meta.Right (kPa)	119.52 ± 44.6 (101.11–137.93)	118.91 ± 55.68 (95.92–141.90)	0.443 ^a^	0.006
Dynamic. P. Average pressure. 4 meta.Left (kPa)	104.70 ± 45.64 (85.86–123.55)	365.68 ± 1259.87 (154.36–885.73)	0.093 ^a^	0.14
Dynamic. P. Average pressure. 5 meta.Right (kPa)	79.60 ± 47.7 (59.91–99.29)	88.13 ± 52.77 (66.35–109.91)	0.158 ^a^	0.08
Dynamic. P. Average pressure. 5 meta.Left (kPa)	62.16 ± 43.67 (44.13–80.19)	89 ± 59.96 (64.25–113.76)	0.002 ^a^	0.24
Dynamic. P. Max. Mdf.Right (kPa)	34.48 ± 37.72 (18.91–50.06)	40.10 ± 46.44 (20.93–59.27)	0.227 ^a^	0.06
Dynamic. P. Max. Mdf. Left (kPa)	32.65 ± 42.99 (14.91–50.40)	35.65 ± 44.9 (17.12–54.19)	0.744 ^a^	0.03
Dynamic. P. Average pressure. Mdf.Right (kPa)	9.97 ± 11.48 (5.23–14.71)	11.12 ± 13 (5.76–16.49)	0.546 ^a^	0.04
Dynamic. P. Average pressure. Mdf.Left (kPa)	9.87 ± 13.93 (4.12–15.62)	10.09 ± 13.49 (4.52–15.66)	0.918 ^a^	0.008
Dynamic. P. Max. Rft.Right (kPa)	321.97 ± 26.19 (236.45–269.99)	317.95 ± 32.7 (304.45–331.45)	0.427 ^b^	0.06
Dynamic. P. Max. Rft.Left (kPa)	340.37 ± 33.90 (311.16–332.78)	323.7 ± 37.66 (308.15–339.24)	0.006 ^b^	0.22
Dynamic. P.Average pressure.Rft.Right (kPa)	132.77 ± 36.54 (196.77–236.53)	135.57 ± 34.43 (121.36–149.78)	0.300 ^b^	0.16
Dynamic. P. Average pressure. Rft.Left (kPa)	140.39 ± 26.84 (117.68–147.85)	131.62 ± 35.98 (116.77–146.47)	0.051 ^b^	0.13
Dynamic. Contact.surfaces.Right (kPa)	106.77 ± 33.52 (129.30–151.47)	103.61 ± 24.13 (93.65–113.57)	0.170 ^a^	0.05
Dynamic. Contact.surfaces.Left (kPa)	101.69 ± 31.09 (92.93–120.60)	100.60 ± 24.63 (90.43–110.77)	0.914 ^a^	0.01
Dynamic. Step.duration.Right (kPa)	767.18 ± 123.00 (88.85–114.53)	755.59 ± 112.14 (709.31–801.88)	0.527 ^a^	0.04
Dynamic. Step.duration.Left (kPa)	764.65 ± 128.56 (711.58–817.72)	762.61 ± 119.07 (713.46–811.76)	0.819 ^b^	0.008
Dynamic. P. Max. Hallux.Right (kPa)	242.50 ± 71.07 (213.16–271.84)	219.47 ± 94.58 (180.42–158.51)	0.174 ^a^	0.13
Dynamic. P. Max. Hallux, Left (kPa)	264.62 ± 68.15 (236.49–292.75)	212.44 ± 74.55 (181.44–243.22)	0.002 ^b^	0.34
Dynamic. P. Average pressure Hallux.Right (kPa)	78.14 ± 40.28 (61.50–94.77)	62.81 ± 40.4 (46.13–79.49)	0.009 ^b^	0.18
Dynamic. P. Average pressure Hallux. Left. (kPa)	83.85 ± 36.35 (68.85–98.86)	58.92 ± 35.63 (44.21–73.63)	0.004 ^a^	0.32

Abbreviations: P, pressure; Max, maximum; 1 meta, first metatarsal; 2 meta, second metatarsal; 3 meta, third metatarsal; 4 meta, fourth metatarsal; 5 meta, fifth metatarsal; Mdf, midfoot; Rft, rearfoot; Kpa, kilopascals; SD, standard deviation. *p* < 0.05 (with a 95% confidence interval) was considered statistically significant; Cohen’s d. ^a^ Wilcoxon signed rank test *p*-value < 0.05 considered statistically significant. ^b^ Paired *t*-test *p*-value considered statistically significant.

## Data Availability

Data will be share by request at evamam03@ucm.es.

## Abbreviations

Pressure, Morton’s extension, Metatarsalgia.

## References

[1] O’Kane C., Kilmartin T.E. (2002). The Surgical Management of Central Metatarsalgia. Foot Ankle Int..

[2] Whitney K.A. (2003). Foot Deformities, Biomechanical and Pathomechanical Changes Associated with Aging Including Orthotic Considerations, Part II. Clin. Podiatr. Med. Surg..

[3] Feibel J.B., Tisdel C.L., Donley B.G. (2001). Lesser Metatarsal Osteotomies. A Biomechanical Approach to Metatarsalgia. Foot Ankle Clin..

[4] Besse J.L. (2017). Metatarsalgia. Orthop. Traumatol. Surg. Res..

[5] van der Leeden M., Steultjens M., Dekker J.H.M., Prins A.P.A., Dekker J. (2006). Forefoot Joint Damage, Pain and Disability in Rheumatoid Arthritis Patients with Foot Complaints: The Role of Plantar Pressure and Gait Characteristics. Rheumatology.

[6] Ko D.Y., Lee H.S. (2013). The Changes of COP and Foot Pressure after One Hour’s Walking Wearing High-Heeled and Flat Shoes. J. Phys. Ther. Sci..

[7] Abdul Razak A.H., Zayegh A., Begg R.K., Wahab Y. (2012). Foot Plantar Pressure Measurement System: A Review. Sensors.

[8] Hähni M., Hirschmüller A., Baur H. (2016). The Effect of Foot Orthoses with Forefoot Cushioning or Metatarsal Pad on Forefoot Peak Plantar Pressure in Running. J. Foot Ankle Res..

[9] Mills K., Blanch P., Chapman A.R., McPoil T.G., Vicenzino B. (2010). Foot Orthoses and Gait: A Systematic Review and Meta-Analysis of Literature Pertaining to Potential Mechanisms. Br. J. Sports Med..

[10] Landorf K.B., Keenan A.M. (2000). Efficacy of Foot Orthoses. What Does the Literature Tell Us?. J. Am. Podiatr. Med. Assoc..

[11] Owings T.M., Woerner J.L., Frampton J.D., Cavanagh P.R., Botek G. (2008). Custom Therapeutic Insoles Based on Both Foot Shape and Plantar Pressure Measurement Provide Enhanced Pressure Relief. Diabetes Care.

[12] Holmes G.B., Timmerman L. (1990). A Quantitative Assessment of the Effect of Metatarsal Pads on Plantar Pressures. Foot Ankle.

[13] Hayda R., Tremaine M.D., Tremaine K., Banco S., Teed K. (1994). Effect of Metatarsal Pads and Their Positioning: A Quantitative Assessment. Foot Ankle Int..

[14] Arias-Martín I., Reina-Bueno M., Munuera-Martínez P.V. (2018). Effectiveness of Custom-Made Foot Orthoses for Treating Forefoot Pain: A Systematic Review. Int. Orthop..

[15] Palomo-Toucedo I.C., González-Elena M.L., Balestra-Romero P., Vázquez-Bautista M.d.C., Castro-Méndez A., Reina-Bueno M. (2023). Pilot Study: Effect of Morton’s Extension on the Subtalar Joint Forces in Subjects with Excessive Foot Pronation. Sensors.

[16] Viehöfer A.F., Vich M., Wirth S.H., Espinosa N., Camenzind R.S. (2019). The role of plantar fascia tightness in hallux limitus: A biomechanical analysis. J. Foot Ankle Surg..

[17] Durrant B., Chockalingam N. (2009). Functional hallux limitus: A review. J. Am. Podiatr. Med. Assoc..

[18] Gordillo-Fernández L.M., Ortiz-Romero M., Valero-Salas J., Salcini-Macías J.L., Benhamu-Benhamu S., García-de-la-Peña R., Cervera-Marin J.A. (2016). Effect by custom-made foot orthoses with added support under the first metatarso-phalangeal joint in hallux limitus patients: Improving on first metatarso-phalangeal joint extension. Prosthet. Orthot. Int..

[19] Patel J., Swords M. (2022). Hallux Rigidus. StatPearls [Internet].

[20] Saíz-Llamosas J.R., Fernández-Pérez A.M., Fajardo-Rodríguez M.F., Pilat A., Valenza-Demet G., Fernández-de-Las-Peñas C. (2009). Changes in neck mobility and pressure pain threshold levels following a cervical myofascial induction technique in pain-free healthy subjects. J. Manip. Physiol. Ther..

[21] Martínez-Jiménez E.M., Losa-Iglesias M.E., Antolín-Gil M.S., López-López D., Romero-Morales C., Benito-de-Pedro M., Calvo-Lobo C., Becerro-de-Bengoa-Vallejo R. (2021). Flexor Digitorum Brevis Muscle Dry Needling Changes Surface and Plantar Pressures: A Pre-Post Study. Life.

[22] Martínez-Jiménez E.M., Losa-Iglesias M.E., Becerro-de-Bengoa-Vallejo R., Díaz-Velázquez J.I., López-López D., Calvo-Lobo C., Rodríguez-Sanz D. (2020). Immediate Effects of Intermittent Bilateral Ankle Plantar Flexors Static Stretching on Balance and Plantar Pressures. J. Manip. Physiol. Ther..

[23] Association W.M. (2013). World Medical Association Declaration of Helsinki: Ethical Principles for Medical Research Involving Human Subjects. JAMA.

[24] Ageberg E., Roberts D., Holmström E., Fridén T. (2005). Balance in Single-Limb Stance in Patients with Anterior Cruciate Ligament Injury: Relation to Knee Laxity, Proprioception, Muscle Strength, and Subjective Function. Am. J. Sports Med..

[25] Van Der Leeden M., Dekker J.H.M., Siemonsma P.C., Lek-Westerhof S.S., Steultjens M.P.M. (2004). Reproducibility of Plantar Pressure Measurements in Patients with Chronic Arthritis: A Comparison of One-Step, Two-Step, and Three-Step Protocols and an Estimate of the Number of Measurements Required. Foot Ankle Int..

[26] Palisano R., Rosenbaum P., Walter S., Russell D., Wood E., Galuppi B. (2008). Development and Reliability of a System to Classify Gross Motor Function in Children with Cerebral Palsy. Dev. Med. Child Neurol..

[27] Yoon S.W. (2015). Effect of the Application of a Metatarsal Bar on Pressure in the Metatarsal Bones of the Foot. J. Phys. Ther. Sci..

[28] Burdock E.I., Fleiss J.L., Hardesty A.S. (1963). A New view of Inter-Observer Agreement. Pers. Psychol..

[29] Portney L.G., Watkins M.P. (2015). Foundations of Clinical Research: Applications to Practice.

[30] Farzadi M., Safaeepour Z., Mousavi M.E., Saeedi H. (2014). Effect of Medial Arch Support Foot Orthosis on Plantar Pressure Distribution in Females with Mild-to-Moderate Hallux Valgus after One Month of Follow-Up. Prosthet. Orthot. Int..

[31] Nathan P.W. (1976). The Gate-Control Theory of Pain. A Critical Review. Brain.

[32] DeLeo J.A. (2006). Basic Science of Pain. J. Bone Jt. Surg. Am..

[33] Hodge M.C., Bach T.M., Carter G.M. (1999). Novel Award First Prize Paper. Orthotic Management of Plantar Pressure and Pain in Rheumatoid Arthritis. Clin. Biomech..

[34] Postema K., Burm P.E.T., Zande M.E.V.D., Limbeek J.V. (1998). Primary Metatarsalgia: The Influence of a Custom Moulded Insole and a Rockerbar on Plantar Pressure. Prosthet. Orthot. Int..

[35] Chang A.H., Harris G.F., Nery J., Shereff M.J., Abu-Faraj Z.U. (1994). Multistep Measurement of Plantar Pressure Alterations Using Metatarsal Pads. Foot Ankle Int..

[36] Kang J.H., Chen M.D., Chen S.C., Hsi W.L. (2006). Correlations between Subjective Treatment Responses and Plantar Pressure Parameters of Metatarsal Pad Treatment in Metatarsalgia Patients: A Prospective Study. BMC Musculoskelet. Disord..

[37] PR C., JS U., GM C. (2000). New Developments in the Biomechanics of the Diabetic Foot. Diabetes Metab Res. Rev..

[38] Morton D.J. (1930). Structural Factors in Static Disorders of the Foot. Am. J. Surg..

[39] Hellstrand Tang U., Zügner R., Lisovskaja V., Karlsson J., Hagberg K., Tranberg R. (2014). Comparison of Plantar Pressure in Three Types of Insole given to Patients with Diabetes at Risk of Developing Foot Ulcers-A Two-Year, Randomized Trial. J. Clin. Transl. Endocrinol..

[40] Martinez-Santos A., Preece S., Nester C.J. (2019). Evaluation of Orthotic Insoles for People with Diabetes Who Are At-Risk of First Ulceration. J. Foot Ankle Res..

[41] Tsung B.Y.S., Zhang M., Mak A.F.T., Wong M.W.N. (2004). Effectiveness of Insoles on Plantar Pressure Redistribution. J. Rehabilit. Res. Dev..

[42] Lee P.Y., Landorf K.B., Bonanno D.R., Menz H.B. (2014). Comparison of the Pressure-Relieving Properties of Various Types of Forefoot Pads in Older People with Forefoot Pain. J. Foot Ankle Res..

[43] Deschamps K., Birch I., Mc Innes J., Desloovere K., Matricali G.A. (2009). Inter- and Intra-Observer Reliability of Masking in Plantar Pressure Measurement Analysis. Gait Posture.

[44] Koenraadt K.L.M., Stolwijk N.M., van den Wildenberg D., Duysens J., Keijsers N.L.W. (2012). Effect of a Metatarsal Pad on the Forefoot during Gait. J. Am. Podiatr. Med. Assoc..

[45] Mueller M.J., Lott D.J., Hastings M.K., Commean P.K., Smith K.E., Pilgram T.K. (2006). Efficacy and Mechanism of Orthotic Devices to Unload Metatarsal Heads in People with Diabetes and a History of Plantar Ulcers. Phys. Ther..

[46] Lima B.N., Lucareli P.R.G., Gomes W.A., Silva J.J., Bley A.S., Hartigan E.H., Marchetti P.H. (2014). The Acute Effects of Unilateral Ankle Plantar Flexors Static-Stretching on Postural Sway and Gastrocnemius Muscle Activity during Single-Leg Balance Tasks. J. Sports Sci. Med..

